# Horticulture therapy affected the mental status, sleep quality, and salivary markers of mucosal immunity in an elderly population

**DOI:** 10.1038/s41598-022-14534-x

**Published:** 2022-06-17

**Authors:** Jui-Ling Shen, Bao-Lien Hung, Shih-Hua Fang

**Affiliations:** 1 Green Healing Academy, Taichung, 406034 Taiwan; 2 Department of Sports Medicine, China Medical University, Taichung, 406040 Taiwan; 3 Institute of Athletics, National Taiwan University of Sport, No. 16, Section 1, Shuangshi Road, Taichung, 404401 Taiwan

**Keywords:** Immunology, Endocrinology, Health care

## Abstract

Horticultural therapy (HT) has been reported to be beneficial to mental and physical health. This study investigated the effects of HT on the psychological status and mucosal immunity of elderly individuals. Twenty-four participants aged 70–93 were recruited from residential facilities and adult day-care services. Six different HT activities were designed and guided by licensed instructors who performed saliva collection and helped the participants complete the questionnaires before and after each activity. The sleep quality scores were collected during the 6 weeks of HT activities. Saliva was collected and analyzed to determine the concentrations of immunoglobulin A (IgA), lactoferrin, chromogranin A (CgA), α-amylase (AA) and total protein (TP). Comparisons of the questionnaire scores between preactivity and postactivity showed that feelings of satisfaction and happiness were significantly enhanced after each activity. In addition, sleep quality was significantly improved after the 6-week course of HT activities. Regarding mucosal immunity, the preactivity IgA and IgA/TP were significantly increased at week 3 and week 6; in addition, the ratio of lactoferrin/TP was significantly decreased at week 6 compared to week 1. The postactivity AA and CgA levels were significantly enhanced at weeks 2, 3 and 5 compared to the corresponding preactivity levels. In conclusions, HT activities significantly improved the happiness, satisfaction, well-being and sleep quality of the elderly. Moreover, mucosal immunity proteins, including IgA, lactoferrin, CgA and AA, were significantly increased.

## Introduction

Horticultural therapy (HT) has been reported to have human health benefits, such as alleviating pressure from stress and anxiety, stabilizing mood changes, establishing self-esteem, increasing the sense of community and improving physical health^1^. The American Horticultural Therapy Association (AHTA) defined HT as an intervention involving indoor or outdoor planting and gardening activities proven to have therapeutic value by reducing stress, decreasing blood pressure and enhancing self-confidence^2^ through stimulating the five senses (visual, sound, taste, tactile and smell) to result in better mental and physical health.

Previous studies demonstrated that elderly people who worked with plants felt an increased sense of responsibility and the chance to solve problems, which reduced the sense of isolation, miserableness, and the risk of cognitive impairment, leading to better psychological health through more social interactions^3^. Empirical studies have shown that physical activity decreases the risk of heart diseases or other illnesses and is beneficial for the elderly^4^. In addition, gardening activity improved the physical functional capacity and motivation of the elderly and enhanced their achievements^5^. Recently, the healing power of nature-based activities for the health and well-being of the elderly was reviewed^6^.

Well-being is a positive feeling and is important for health maintenance. Although there were cultural differences in relationship-relevant happiness, positive emotions generally present as a valuable and meaningful life^7^. A review clearly demonstrated the importance and benefits of using HT for improving well-being and sleep quality in dementia care^8^. Two other studies have reported that sensory stimulation of dementia patients could ameliorate mood disturbances and behavioral problems and increase mental well-being and quality of life^9,10^. Later, a meta-analysis proved that HT had a positive effect on cognitive function not only in randomized controlled trials but also in well-designed controlled trials^11^.

On the other hand, it is well known that saliva contains various immune defense proteins, such as immunoglobulin A (IgA), lactoferrin, chromogranin-A (CgA) and α-amylase (AA), which are potentially important barriers against bacterial or viral damage. AA is not only a digestive enzyme that breaks down starch but is also important in mucosal immunity. In addition, AA has been proposed as a marker of the activation of the sympathoadrenal medullary system (SAM)^12^. CgA exhibits antibacterial and antifungal activities to increase host defense^13^ and is also a sensitive and quantitative index of the sympathetic nervous system (SNS)^14^.

Because saliva collection is a convenient, noninvasive sampling method, it is practical to investigate the levels of salivary proteins associated with the risk of upper respiratory tract infection (URTI)^15^. Several reviews in the literature have focused on the emotional benefits of HT, such as reducing stress, stabilizing mood, and increasing the sense of spirituality and enjoyment^16–18^. Taiwan is already an aging society, and it will become a super-aged society by 2025^19^. The gardening activities might exhibit the better healing effect on the elderly, which can be used as a reference when arranging different courses in the residential facilities and adult day-care services centers in the future. To date, few studies have investigated the effects of HT on mucosal immunity. We wondered whether these positive emotions induced by engaging in HT activities would affect the mucosal immunity of the elderly. Therefore, this study investigated the benefits of different types of HT activities on psychological status by questionnaires and furthermore evaluated the potential biomarkers of mucosal immunity in the saliva of elderly individuals during HT activities.

## Materials and methods

### Subjects and ethical statement

Twenty-four participants were recruited from residential facilities (6/24) and adult day-care services (18/24) in Taichung, Taiwan. The participants have to meet the following criteria: The elders can participate in gardening activities with the assistance of caregivers after communication. Participants were excluded if they were bedridden or unable to control speech. Based on a physician-based interview using the Clinical Dementia Rating (CDR) scale rating of 0–3 (0 = none; 0.5 = questionable; 1 = mild; 2 = moderate; 3 = severe)^20^, 15 among 24 participants were diagnosed as 0.5–3. The other participants without dementia did not have other diseases. They were sent and cared in adult day-care services via family members who were wheelchair-bound or too old to live alone. The caregiver helps fill in the questionnaire according to the condition of the elderly who unable fill in it.

The adequate sample size was calculated using G*power version 3.1.9.7 software (Heinrich-Heine-Universität, Düsseldorf, Germany). A previous study suggested that a sample size of 18 would yield a power of 0.8 (with two-tailed alpha of 0.05), which is sufficient to detect effects of HT on mucosal immunity^21^. The sample size of 24 participants in this investigation was sufficient. All experimental procedures and protocols were approved by the Institutional Human Ethics Committee of Jen-Ai Hospital, Taichung, Taiwan (#109-05). Each participant was fully informed of all the potential risks and experimental procedures, and written informed consent was obtained. All experiments were performed in accordance with relevant guidelines and regulations.

### Study design

Six different themes of HT were designed and guided by the same instructor certified by the Asia Pacific Association of Therapeutic Horticulture (APATH). The HT activities included (1) planting vegetables, (2) making a rosemary wreath, (3) designing a healing garden, (4) packaging a jar of black date vinegar, (5) arranging a romantic rose basket, and (6) a garden party and all were carried out in the day care center. The objectives and contents of each program are described in Table 1. The instructor helped the participants collect saliva and complete the questionnaire before (at 2:00 pm) and after (at 3:00 pm) every course. Saliva was analyzed to determine the concentrations of IgA, lactoferrin, CgA, AA and total protein (TP).

**Table 1:** The objectives and contents of the six horticultural activity sessions.

Unit	Topic	Objectives	Contents
1	Planting vegetables	To increase social connection To build up confidence and increase interest	Seeding and watering every day Discussing the growth of vegetables or just chatting together during watering time Harvesting and tasting vegetables with the help of the caregiver
2	Making a rosemary wreath	To increase hand–eye coordination To increase the participants' sense of aesthetic appreciation of their surroundings, achievement, and satisfaction	Smelling the fragrance of rosemary Using fingers to wrap the rosemary into a small wreath with iron wire
3	Designing a healing garden	To stimulate a subjective decision to make a small garden To enhance space perception ability and imagination	Using a potted plant, small stones, and decorations to design one’s own healing garden Fixing the position of the arrangement with glue
4	Packaging a jar of black date vinegar	To enhance the participants' sense of aesthetic appreciation of their surroundings and curiosity To initiate positive motivation toward expectations	Putting the black dates into a jar, pouring the vinegar, sealing the package and labeling Soaking the products for 3 months
5	Arranging a romantic rose basket	To encourage to design To increase the participants' sense of aesthetic appreciation of their surroundings and happiness	Smelling the fragrance of roses and cutting into different lengths Designing and inserting the roses into a green sponge in a small basket
6	Garden party	To enjoy the sunshine and the breeze in the garden	Sitting or walking around all products from previous horticultural activities Sharing feelings from the favorite horticultural session

### Questionnaire measurements

#### Sleep assessment

The Taiwanese version of the Pittsburgh Sleep Quality Index (PSQI-T)^22^ was used to assess the sleep quality of participants before and after the 6-week activities. The reliability of the PSQI-T, i.e., the Cronbach’s alpha for the PSQI, was 0.79. The test–retest reliability was 0.91, and the validity of the sensitivity and specificity were excellent.

#### Satisfaction with Life Scale (SWLS)

A questionnaire of 5 specifically worded items with scores ranging from 1 to 7 for evaluating satisfaction with life. It has good validity and reliability within the SWLS-Chinese version^23^. The scores range from 5 to 35, and higher scores indicate greater life satisfaction.

#### Warwick–Edinburgh Mental Well-Being Scale (WEMWBS)

A questionnaire containing 14 specifically worded items with scores ranging from 1 to 5 for evaluating psychological well-being, which was constructed by the researchers at the Universities of Warwick and Edinburgh. The scores range from 14 to 70, and higher scores indicate better mental well-being. The Chinese SWEMWBS has high internal consistency and reliability against accepted criteria^24^.

#### Happiness and Life Satisfaction (HLS)

Defined by commonly reliable subjective well-being, which includes main feelings, contentment and meaning^25^. All data were converted to scores ranging from 0 to 10 for the happiness index (10 = very happy, 5 = moderately happy, 0 = extremely sad).

### Saliva collection and assay

Saliva was collected with a cotton swab into special sampling tubes (Salivettes; Sarstedt, Numbrecht, Germany). Briefly, the participants sat, and their mouths were rinsed with 30 ml of drinking water before sample collection. Participants chewed a piece of cotton for 5 min and then put into the tubes. The tubes were then centrifuged at 1500×*g* for 10 min to obtain clear saliva, which was stored immediately at − 80 °C until assay. The concentrations of IgA, lactoferrin, CgA and TP were determined using ELISA kits (Cozart Bioscience Ltd, Oxfordshire, UK). AA activity was determined using a kinetic reaction assay kit (Salimetrics LLC, State College, PA, USA) according to the manufacturer’s instructions. All samples were measured in triplicate. The intra-assay coefficients of variation (CVs) for the measurements of IgA, lactoferrin, AA, CgA and TP activity were 5%, 4%, 4%, 5% and 4%, respectively.

### Data analysis

All data are expressed as the mean ± standard deviation (SD). One-way repeated-measures ANOVA with a Bonferroni post hoc test was used to determine the significant differences in the changes in mucosal immunity-related proteins at each time point. Statistical comparisons between the preactivity and postactivity questionnaires were analyzed using paired t-test. Significant differences were set at *p* < 0.05.

## Results

### Psychological state analysis

Twenty-four participants, including 7 men and 17 women, attended the 6-week HT activities, and the average ages were 81.71 ± 9.01 (70–93) and 80.65 ± 5.22 (72–93), respectively. Based on comparing the scores of SWLS, WEMWBS and HLS between preactivity and postactivity, dramatically improved feelings of satisfaction and happiness were found after each activity (*p* < 0.001, Table 2).

**Table 2:** The SWLS, WEMWBS and HLS scores preactivity and postactivity in each week.

Week		SWLS			WEMWBS			HLS		
		Mean ± SD	T	*p* value	Mean ± SD	T	*p* value	Mean ± SD	T	*p* value
1	Pre	21.73 ± 5.59	6.223	< .001	44.05 ± 10.77	8.056	< .001	5.73 ± 1.72	8.120	< .001
	Post	27.68 ± 5.12			62.23 ± 5.72			8.50 ± 1.54		
2	Pre	22.17 ± 7.12	5.743	< .001	45.29 ± 12.09	6.493	< .001	6.00 ± 1.82	7.287	< .001
	Post	27.38 ± 6.19			62.79 ± 7.39			8.63 ± 1.58		
3	Pre	21.33 ± 6.78	7.013	< .001	41.71 ± 12.01	9.306	< .001	5.65 ± 2.04	8.066	< .001
	Post	27.33 ± 7.28			58.96 ± 13.98			8.65 ± 1.50		
4	Pre	23.21 ± 3.84	7.920	< .001	45.83 ± 7.76	12.85	< .001	5.67 ± 1.88	8.008	< .001
	Post	30.00 ± 4.20			64.83 ± 6.14			8.75 ± 1.70		
5	Pre	18.68 ± 5.78	7.235	< .001	40.58 ± 14.15	8.357	< .001	5.29 ± 2.24	7.224	< .001
	Post	29.68 ± 3.21			65.54 ± 6.74			8.88 ± 1.60		
6	Pre	22.38 ± 5.97	6.022	< .001	53.21 ± 13.89	4.105	< .001	5.96 ± 1.57	8.027	< .001
	Post	30.54 ± 5.60			65.17 ± 6.25			9.21 ± 1.28		

SWLS, Satisfaction with Life Scale; WEMWBS, Warwick–Edinburgh Mental Well-being Scale; HLS, Happiness and Life Satisfaction.

### Sleep quality analysis

**Figure 1:**
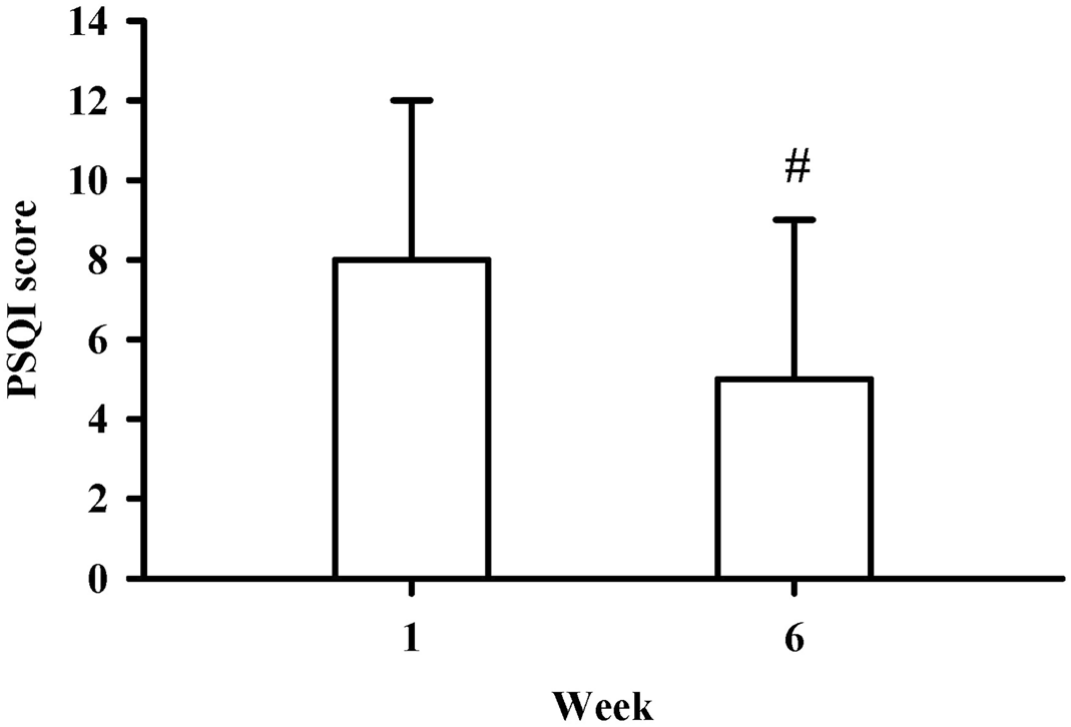
The PSQI was measured before and after 6 weeks of activity. ^#^*p* < 0.01 compared to week 1. Data are presented as the mean ± SD.

In addition, comparison of the PSQI scores between week 6 and week 1 revealed significant improvement in sleep quality after the 6-week HT activities (7.92 ± 3.75 vs. 5.21 ± 3.60, *p* < 0.01, Fig. 1).

### Salivary immune protein measurements

**Figure 2:**
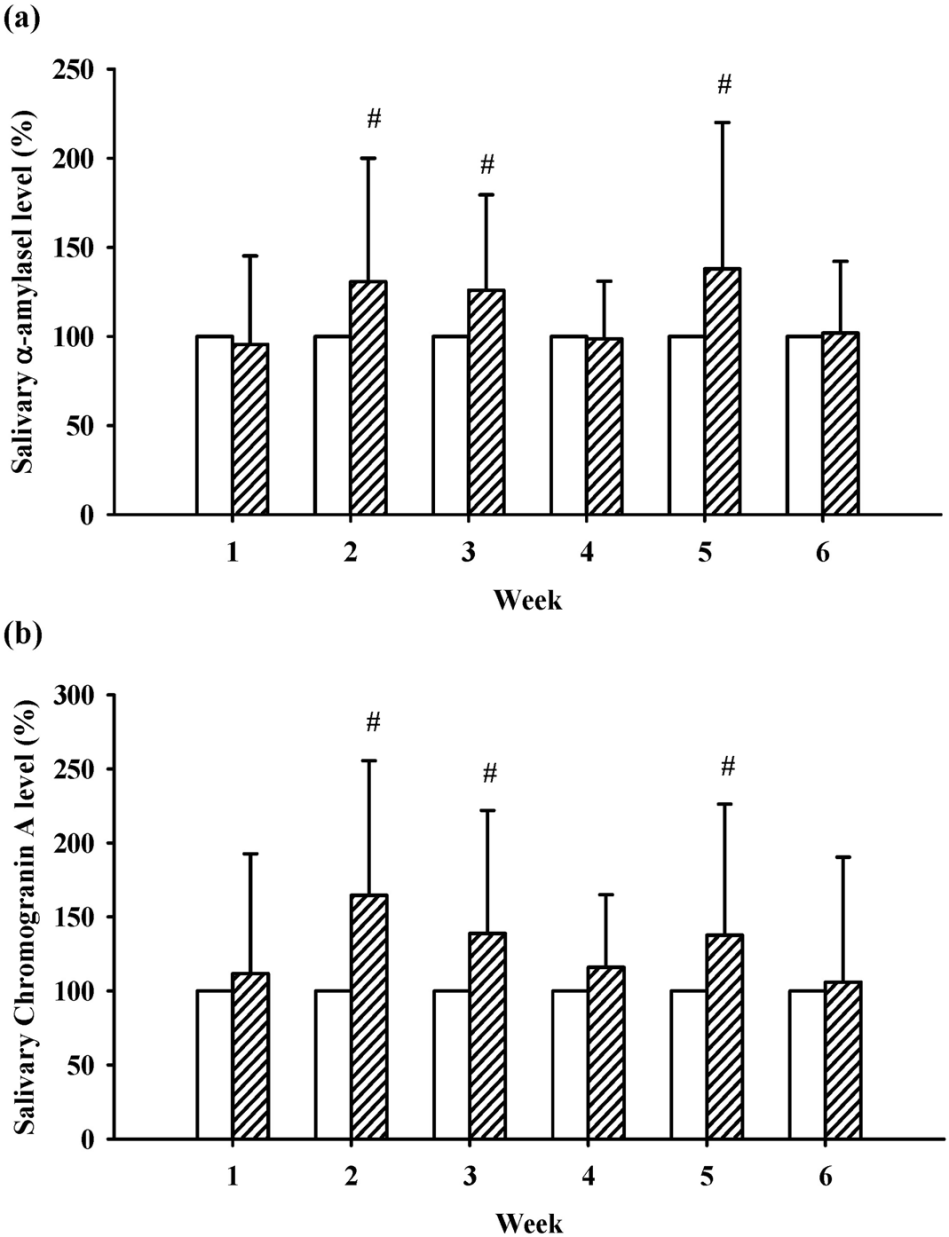
The preactivity and postactivity relative levels of α-amylase (**a**) and chromogranin A (**b**) in saliva at different weeks. The empty bar represents preactivity. The slashed bar represents postactivity. ^#^*p* < 0.01 compared to preactivity at each week. Data are presented as the mean ± SD.

As shown in Table 3, the preactivity levels of IgA and IgA/TP were significantly increased at week 3 (286.86 ± 375.56 vs. 101.54 ± 83.83, *p* < 0.05 and 100.12 ± 110.43 vs. 34.36 ± 26.83, *p* < 0.05) and week 6 (612.95 ± 531.33 vs. 101.54 ± 83.83, *p* < 0.001 and 202.25 ± 161.32 vs. 34.36 ± 26.83, *p* < 0.001), respectively, when compared to week 1. In addition, the ratio of lactoferrin/TP was significantly increased at week 6 (7.92 ± 3.75 vs. 5.21 ± 3.60, *p* < 0.05). The postactivity AA and CgA levels relative to the preactivity levels (set as 100%) were significantly enhanced at weeks 2, 3 and 5 (*p* < 0.05, Fig. 2).

**Table 3:** Changes in immunity-related proteins in saliva during 6 weeks of HT activity.

	Week 1	Week 3	Week 6
IgA (µg/ml)	101.54 ± 83.83	286.86 ± 376.56^a^	612.95 ± 531.33^aaa,b^
Lactoferrin (µg/ml)	33.29 ± 35.41	41.15 ± 51.49	46.99 ± 64.35
TP (mg/ml)	3.42 ± 1.43	2.91 ± 1.11	3.49 ± 1.99
IgA/TP (µg/mg)	34.36 ± 26.83	100.12 ± 110.43^a^	202.25 ± 161.32^aaa,b^
Lactoferrin/TP (µg/mg)	7.28 ± 6.55	11.90 ± 14.94	13.33 ± 14.75^a^

Data are expressed as mean ± SD.

TP, total protein expressed as bovine serum albumin level.

^a^*p* < 0.05, ^aaa^*p* < 0.001 compared to Week 1.

^b^*p* < 0.05 compared to Week 3.

## Discussion

The results of this study indicated that the subjective feelings of life satisfaction, well-being and happiness of the elderly were enhanced after each of the six HT activities. In addition, the sleep quality of the elderly was improved after the 6-week HT program. There are many factors that can influence sleep quality^26^. This study is to demonstrate that the elderly participated in the HT activity sessions and appreciated of themselves finished products for six consecutive weeks. Furthermore, the levels of sympathetic nerve activation-related immune defense proteins, i.e., AA and CgA, were immediately enhanced at weeks 2, 3 and 5. Moreover, the ratios of IgA/TP and lactoferrin/TP were increased after the 6-week HT program. In brief, our qualitative and quantitative results clearly indicated that the designed HT programs were effective at increasing positive mental emotions, sleep quality and mucosal immunity of the elderly. Among the 24 participants in this study, although 15 of them were diagnosed with different degrees of dementia, we observed that they all enjoyed HT activities regardless of the stage of dementia. Previous studies reported that HT had mental benefits for elderly individuals without and with mild or moderate dementia^27,28^, which was consistent with our findings.

Psychological well-being and health are closely linked at older ages. Previous studies showed that HT could increase many psychosocial health factors and the quality of life in older housing care residents^29–31^. Obviously, the increase in happiness was related to other relevant variables, including well-being, personal efficacy or participation in community life. The well-being is sometimes moved by the moment, or it is a feeling accumulated over a long period of time. A methodical review of 27 observational studies assumed that elderly individuals increased their pleasure from observing or doing something in the natural environment, which might have a positive effect on their well-being and sense of usefulness^32^. The mean SWLS scores before each activity in this study were lower than those obtained in previous studies of Europeans^33,34^, which we interpreted to mean that the elderly people in residential facilities or adult day-care services were not happy. A previous study stated that a change of 3 points in the WEMWBS represents the minimum clinically important difference^35^. In this study, the WEMWBS scores were increased over 11 points after each HT activity, and the SWLS scores after each activity were also enhanced dramatically. Moreover, the happiness index of participants after each activity was significantly higher than 6.75, the average value of most people^36^. In addition, consistent with our finding showing that the elderly had better sleep quality after HT activities, previous reports suggested that it was related to nature, which exerted beneficial effects on positive pleasure, sleep quality, stress release and mood state^37^.

More importantly, this is the first study to report significant increases in the levels of IgA and lactoferrin after HT intervention, which opens up a new approach for researchers in this field. Salivary IgA is the principal antibody in the mucosal system and can prevent upper respiratory diseases. After six courses, the elderly had higher levels of IgA and IgA/TP at weeks 3 and 6. In addition, lactoferrin/TP was increased at week 6. Several studies have reported that forest bathing decreased cardiovascular disease-related biomarkers, blood pressure, urinary adrenaline and cortisol in serum and saliva^38–40^. Furthermore, a study demonstrated that following a 6-month HT program, T-cell exhaustion and inflammation were reduced in community-dwelling older adults^41^. These facts indicated the need for continued HT practices to improve hormone and immune factors, which in turn improved health conditions. We suggest that salivary proteins such as IgA and lactoferrin could be used in future studies to evaluate the beneficial effects of HT intervention on the immune system.

CgA and AA are known psychophysiological stress markers, and we suspected that CgA and AA would be reduced after HT intervention. However, contrary to our expectation, the levels of CgA and AA were increased at weeks 2, 3 and 5. Previous studies have reported that significant increases in CgA were associated with psychological relaxation and positive stimulation, such as experiencing woodland walking and short leisure trips or resulting from laughter when feelings were uplifted^42–44^. In addition, there was a good correlation between AA and catecholamines^45^. Moreover, a recent review discussed the pivotal role of the SNS in the crosstalk with the immune system^46^. Happiness has been characterized by hyperactivating the SNS. Therefore, HT-induced happiness and satisfaction are associated with SNS activation; nevertheless, at present, we cannot elucidate the underlying mechanism. Similar to the defensive effects of IgA and lactoferrin, mucosal immune function might also be enhanced by increased levels of CgA and AA.

Finally, this study still had several limitations of the design. First, the participants had different CDR statuses but participated in the same activity. Second, the present sample size from residential facilities or adult day-care services was not sufficient. Thus, we were not able to compare the different responses among elderly individuals with different dementia statuses. Expanding the sample size to different levels of CDR would strengthen the design of HT programs for evaluating elderly individuals with dementia. Third, the changes in the participants’ social communications were not fully captured by the design and measurement during the HT sessions. Further study could be designed to monitor the changes in their emotional expression. Importantly, the age structure of the elderly population is rapidly aging, and the health of the elderly is growing important. Hopefully, the benefits of HT to the elderly can be found to further include as one of the rehabilitation activities supported by Taiwan's National Health Insurance in the future.

In conclusion, after the 6-week HT activities, the happiness, life satisfaction, mental well-being and sleep quality of the elderly were significantly improved. Moreover, the mucosal immunity markers IgA and IgA/TP were increased at week 3 and week 6, whereas lactoferrin/TP was increased at week 6, implying that these types of activities can be a priority in the design of gardening activities for the elderly in the future.
